# Lytic *Escherichia* phage OSYSP acts additively and synergistically with gaseous ozone against *Escherichia coli* O157:H7 on spinach leaves

**DOI:** 10.1038/s41598-023-36815-9

**Published:** 2023-07-03

**Authors:** Mustafa Yesil, David R. Kasler, En Huang, Ahmed E. Yousef

**Affiliations:** 1 Department of Food Science and Technology, The Ohio State University, 2015 Fyffe Road, Columbus, OH 43210 USA; 2 Department of Microbiology, The Ohio State University, 105 Biological Sciences Building, 484 W. 12th Ave, Columbus, OH 43210 USA; 3 Present Address: Department of Environmental Health Sciences, University of Arkansas for Medical Sciences, Little Rock, AR 72205 USA

**Keywords:** Bacteriophages, Bacteriology

## Abstract

Bacteriophage and gaseous ozone are evolving as meritorious alternatives to conventional sanitizers in food postharvest applications. Here, we investigated the efficacy of sequential treatments of a lytic bacteriophage and gaseous ozone, during vacuum cooling of fresh produce, against *Escherichia coli* O157:H7. Spinach leaves were spot-inoculated with 10^5^–10^7^ CFU g^−1^* E. coli* O157:H7 B6-914 and treated with *Escherichia* phage OSYSP spray (10^9^ PFU g^−1^), gaseous ozone, or their combination. Vacuum cooling, which preceded or followed phage application but ran concomitantly with ozone treatment, was performed in a custom-made vessel at the following process sequence: vacuum to 28.5 in. Hg, vessel pressurization to 10 psig with gas containing 1.5 g ozone/kg gas-mix, holding for 30 min, and vessel depressurization to ambient pressure. Bacteriophage or gaseous ozone inactivated *E. coli* O157:H7, applied at different initial populations on spinach leaves, by 1.7–2.0 or 1.8–3.5 log CFU g^−1^, respectively. At the high inoculum levels tested (7.1 log CFU g^−1^), sequential treatments of phage and ozone reduced *E. coli* O157:H7 population by 4.0 log CFU g^−1^, but when treatment order was reversed (i.e., ozone followed by bacteriophage), the combination synergistically decreased pathogen’s population on spinach leaves by 5.2 log CFU g^−1^. Regardless the antibacterial application order, *E. coli* O157:H7 populations, applied initially at ~ 10^5^ CFU g^−1^, were reduced below the enumeration method’s detection level (i.e., < 10^1^ CFU g^−1^). The study proved that bacteriophage–ozone combination, applied in conjunction with vacuum cooling, is a potent pathogen intervention strategy in fresh produce post-harvest applications.

## Introduction

Contamination with Shiga toxin-producing *Escherichia coli* (STEC) is a great safety concern in the fresh produce industry^1^. This pathogen is an important cause of foodborne disease outbreaks associated with *E. coli* in the United States^2^. *E. coli* strains are largely harmless members of the gastrointestinal microbiota; these even play a beneficial role in digestion, production of essential vitamins, and elimination of harmful microorganisms via competitive exclusion^3,4^. However, some strains are pathogenic, particularly the members of STEC group such as *E. coli* O157:H7^5^. During the period between 2017 and 2021, *E. coli* O157:H7-contaminated leafy greens have caused 529 illnesses, 243 hospitalizations, and 7 deaths in the United States^6^ and the pathogen remains one of the main causes of recurring fresh produce-related disease outbreaks.

Leafy vegetables are grown in open fields in proximity to soil and its organic matters, a condition which causes product contamination and decreases the efficacy of disinfection process with conventional sanitizers^7^. Spraying fresh produce with chlorinated water immediately after harvest is done to compensate for water loss during vacuum cooling^8^ and to minimize the adaptation of pathogens to product environment^9^. Fresh produce is rapidly pre-cooled (e.g., through vacuum-cooling) after harvesting to remove the field heat, thus, slowing down the respiration rate of plant tissues^10^. In packing facilities, fresh produce is often washed with sanitizer-containing water. Chlorine is the most commonly used sanitizer due to its low cost, ease of implementation, and ability to minimize cross contamination between batches of fresh produce^11^. However, concerns have been raised due to the overuse of chlorine and possible formation of carcinogenic side products in chlorinated wash water^9^. There is also a growing awareness of the need to sustain water resources, which are used liberally in fresh produce pre- and post-harvest operations^12^. It is, therefore, plausible to explore new approaches for decontaminating fresh produce.

Alternative postharvest pathogen interventions, such as the application of bacteriophage and ozone, have been the subject of many recent studies^13,14^. Bacteriophages are found in high numbers in host environment, making them relatively easy to isolate and logical to test in food applications^15^. Leafy greens are suitable products for phage application because their pH supports phage viability and action^16^. Application of single lytic phage, cocktail of phages, or combination of phage with other antimicrobial agents significantly inactivated various microorganisms, in comparison with untreated controls^13,16,17^. Before use in food applications, promising phages must undergo safety and efficacy evaluations, including the assessment of lytic life cycle, host-range, propagation in non-pathogenic hosts, compatibility with the food environment, and genetic characterization to confirm the absence of toxic and allergenic determinants in their genomes^18,19^. Ten unique commercial phage preparations targeting foodborne pathogens were affirmed as Generally Recognized as Safe (GRAS) by FDA between 2006 and 2018^20^. *Escherichia* phage OSYSP, a non-commercial lytic-phage, was previously isolated from environmental samples. When cut green pepper and spinach leaves were treated with this phage, populations of *E. coli* O157:H7 EDL933 decreased by 2.4–3.0 and 3.4–3.5 log CFU/g, respectively, during 72 h storage^17^. The safe use of this phage was also assessed through complete genome analysis, which confirmed the absence of microbial toxin-encoding genes^21^.

Ozone gas is a highly oxidizing sanitizer, which when used at optimum levels results in high microbial reduction (> 3 log CFU g^−1^ reduction), compared to the untreated control, without damaging the visual quality of fresh produce^22^. Ozone has a broad antimicrobial activity, and it is more stable in the gaseous than in the aqueous phase^23^. Due to its short half-life, ozone is produced on-site from oxygen and used as an antimicrobial agent under controlled treatment settings^23^. Similar to other oxidizing agents, using ozone requires taking safety precautions; improper use may pose risks to both food processing equipment and personnel exposed to it^24^. Ozone decomposes readily to oxygen without leaving chemical residues^25^; hence, the use of ozone is a desirable approach for fresh produce decontamination.

The perishability of fresh produce necessitates the use of hurdle techniques to effectively control pathogens without causing product quality deterioration. Applying the bacteriophage at higher concentration (PFU ml^−1^) than that of its host (CFU g^−1^) could overwhelm the host bacterium response to virus infections, and results in early lysis of bacterial cells. This well-known mechanism, in which host cells are lysed by bacteriophage without its replication, is called “lysis from without”^26^. Internalization of pathogenic microorganisms into the leafy green tissues is well documented^27^. These internalized pathogens would be less accessible to the aqueous bacteriophage application, compared to the gaseous ozone treatment. Enhanced efficacy of gaseous ozone was reported when applied subsequent to vacuum due to greater penetration potential into internal parts of the treated food, e.g., shell eggs^28^. As opposed to host specificity of bacteriophages, gaseous ozone has a broader antimicrobial capacity^23^ and may inactivate both surface and internalized microbial contaminants. Therefore, it was hypothesized that combining bacteriophage and gaseous ozone in fresh produce decontamination would be a comprehensive intervention strategy that minimizes pathogen risk and product quality deterioration. Thus, the goal of the study was to test the efficacy of *Escherichia* phage OSYSP and gaseous ozone, individually or in combination, in inactivating *E. coli* O157:H7 inoculated on spinach leaves. The specific objectives of this research were: (1) to assess whether the postharvest bacteriophage application on spinach leaves followed by gaseous ozone treatment during vacuum cooling enhances the inactivation of *E. coli* O157:H7, (2) to determine if changing the order of antimicrobial application changes the level of inactivation of *E. coli* O157:H7, and (3) to evaluate the effect of pathogen inoculum size on the level of inactivation by phage, gaseous ozone, and combination treatments.

## Results

### Application of *Escherichia* phage OSYSP to control *Escherichia coli* O157:H7 B6-914 on spinach leaves

**Figure 1:**
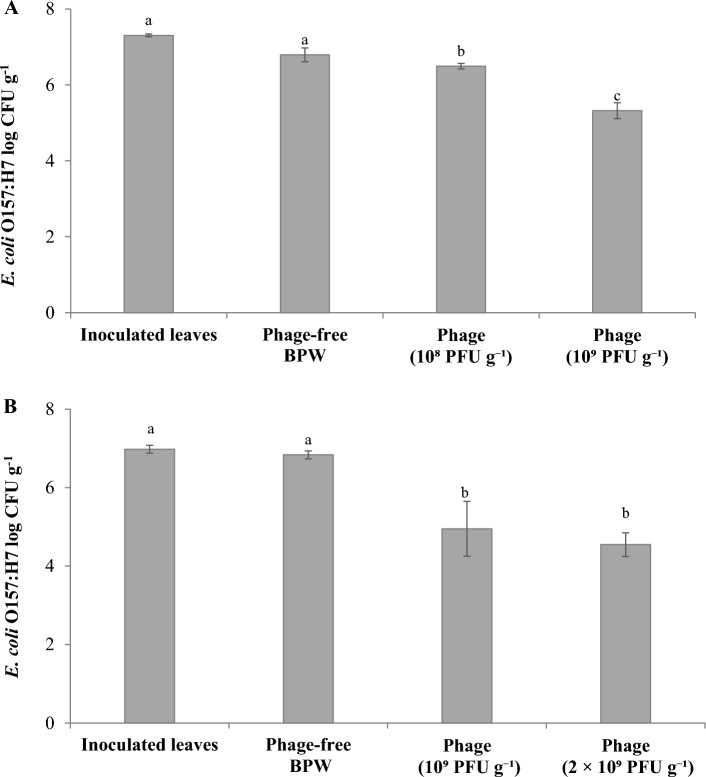
Changes in the populations of *Escherichia coli* O157:H7 B6-914, spot-inoculated on spinach leaves, when *Escherichia* phage OSYSP suspensions were applied at different concentrations and volumes. (**A**) Effect of phage concentrations. (**B**) Effect of a single and repeated bacteriophage spray (doubling the spray volume). Average log reductions were obtained from at least 3 independent treatments. Different letters on bars represent significant difference (*P* < 0.05). Error bar indicates ± standard error of the mean. *Escherichia* phage OSYSP spray was applied at 10^8^, 10^9^, or 2 × 10^9^ PFU g^−1^ spinach.

The effect of phage titer against high initial load of *E. coli* O157:H7 B6-914 (7.3 log CFU g^−1^) was assessed. Applying bacteriophage suspensions as a spray at 10^8^ and 10^9^ PFU g^−1^ on spinach leaves reduced *E. coli* O157:H7 counts by 0.3 and 1.5 log CFU g^−1^, respectively, compared to the spray with phage-free buffered peptone water (BPW) (Fig. 1A). Phage application was further assessed to determine the effect of repeating (doubling) phage spray, which completely covered the spinach leaves, on microbial reductions. At the initial load of 7.0 log CFU g^−1^ *E. coli* O157:H7 B6-914, phage applications significantly (*P* < 0.05) reduced *E. coli* O157:H7 B6-914 counts by 1.9 and 2.3 log CFU g^−1^ at non-repeating (10^9^ PFU g^−1^) and repeating (2 × 10^9^ PFU g^−1^) phage spray applications, respectively, compared to the phage-free BPW spray; however, microbial reductions in these two treatments were not significantly different (*P* > 0.05) from each other (Fig. 1B).

### Sequential application of bacteriophage and gaseous ozone against *Escherichia coli* O157:H7 B6-914 on spinach leaves

**Figure 2:**
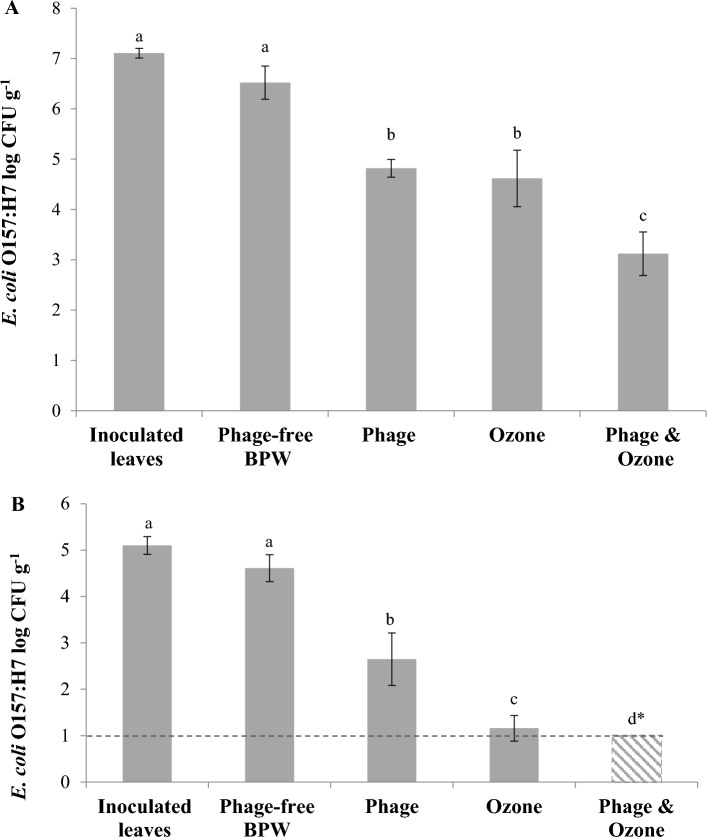
Changes in *Escherichia coli* O157:H7 B6-914 populations applied on spinach leaves at high and low inoculum levels and treated with *Escherichia* phage OSYSP, gaseous ozone, and sequential treatments of phage followed by gaseous ozone. (**A**) High inoculum size; 10^7^ CFU g^−1^. (**B**) Low inoculum size; 10^5^ CFU g^−1^. *Dotted line shows the limit of detection. Average log reductions were obtained from at least 3 independent treatments. Different letters on bars represent significant difference (*P* < 0.05). Error bar indicates ± standard error of the mean. *Escherichia* phage OSYSP spray was applied at 10^9^ PFU g^−1^ spinach.

Treatments of pathogen-inoculated spinach leaves included the following (Fig. 2): (a) phage-untreated (control 1); (b) spray with phage-free BPW (control 2); (c) spray with phage suspension; (d) spray with phage followed by treatment with gaseous ozone; and (e) spray with phage-free-BPW followed by gaseous ozone treatment. All gaseous ozone treatments were done in conjunction with vacuum cooling, as described in the methodology sections. Additional experiments were conducted to assess the effect of pathogen inoculum size (approximately 5 and 7 log CFU g^−1^) on its inactivation by the previously described treatments. At 7.1 log CFU g^−1^ of inoculum level, phage-free BPW spray decreased the target pathogen population by a 0.6 log CFU g^−1^, compared to the inoculated untreated control samples (Fig. 2A), but this reduction was not statistically significant (*P* > 0.05). Treating the artificially contaminated spinach leaves with the phage suspension or gaseous ozone significantly (*P* < 0.05) decreased *E. coli* O157:H7 B6-914 counts by 1.7 and 1.9 log CFU g^−1^, respectively, compared to the phage-free BPW. However, mean log reductions from phage and ozone treatments were not significantly different (*P* > 0.05). Sequential application of phage and gaseous ozone reduced the viable *E. coli* O157:H7 B6-914 counts by 3.4 log CFU g^−1^, when compared with the phage-free BPW. Therefore, at the high inoculum of *E. coli* O157:H7 B6-914, phage, ozone and their sequential applications significantly inactivated *E. coli* O157:H7 B6-914 on spinach leaves, and the sequential application caused additive antimicrobial effect, and was significantly (*P* < 0.05) more effective than the individual phage or ozone treatment.

Similarly, the previous experiments were repeated on spinach leaves inoculated with *E. coli* O157:H7 B6-914 at 5.1 log CFU g^−1^ inoculation level (Fig. 2B). The population of *E. coli* O157:H7 B6-914 recovered from the spinach leaves treated with BPW (control) was insignificantly (*P* > 0.05) different from that recovered on the inoculated untreated leaves. At this *E. coli* low inoculum level, phage and gaseous ozone treatment, applied separately, significantly reduced bacterial counts on spinach leaves by 2.0 and 3.5 log CFU g^−1^, respectively, compared to the phage-free BPW spray. *E. coli* O157:H7 B6-914 reduction with the gaseous ozone treatment was significantly (*P* < 0.05) higher than that for the phage treatment. The sequential application of phage OSYSP followed by a gaseous ozone treatment produced the highest inactivation and reduced the *E. coli* O157:H7 B6-914 count below the enumeration method’s limit of detection, i.e., < 1 log CFU g^−1^. Comparison of inactivations from two different inoculum levels showed that the efficacy of phage, ozone, and combination treatments (phage followed by gaseous ozone) were significantly higher (*P* < 0.05) at the lower inoculum level than those obtained at the high inoculum level.

### Application of gaseous ozone followed by bacteriophage against *Escherichia coli* O157:H7 B6-914 on spinach leaves

**Figure 3:**
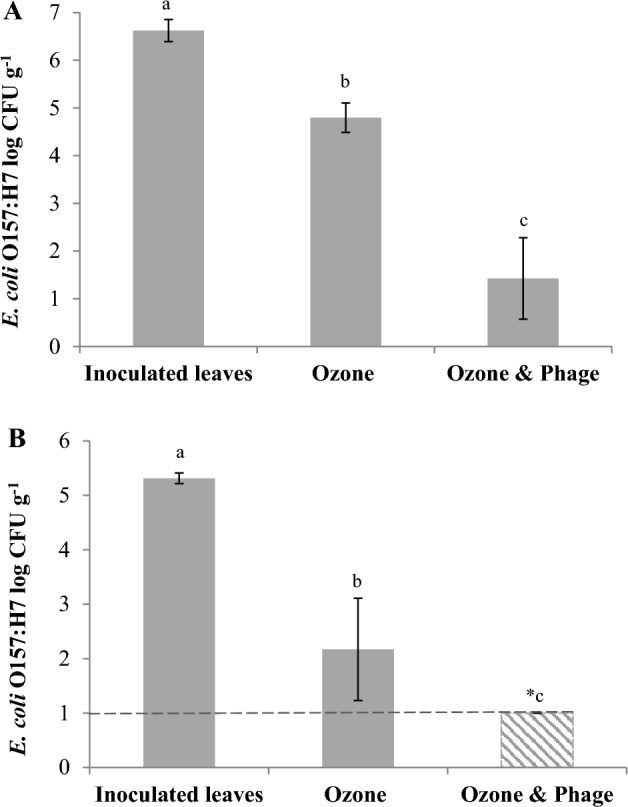
Changes in *Escherichia coli* O157:H7 B6-914 populations applied on spinach leaves at high and low inoculum levels and treated with *Escherichia* phage OSYSP, gaseous ozone, and sequential treatments of gaseous ozone followed by *Escherichia* phage OSYSP. (**A**) High inoculum size; 10^7^ CFU g^−1^. (**B**) Low inoculum size; 10^5^ CFU g^−1^. *Dotted line shows the limit of detection. Average log reductions were obtained from at least 3 independent treatments. Different letters on bars represent significant difference (*P* < 0.05). Error bar indicates ± standard error of the mean. *Escherichia* phage OSYSP spray was applied at 10^9^ PFU g^−1^ of ozone-treated spinach.

Antimicrobial application order and inoculum level of pathogen were investigated (Fig. 3). Inoculated leaves were treated with gaseous ozone first, then followed by application of the bacteriophage. At 6.6 log CFU g^−1^ inoculation level, gaseous ozone and sequential treatments of gaseous ozone followed by bacteriophage significantly (*P* < 0.05) decreased viable *E. coli* O157:H7 B6-914 counts by 1.8 and 5.2 log CFU g^−1^, respectively (Fig. 3A). At 5.3 log CFU g^−1^ inoculation level, reductions were more pronounced (Fig. 3B). At this lower inoculum level, ozone application alone reduced the *E. coli* populations significantly (*P* < 0.05) by 3.1 log CFU g^−1^. No survivors were detected in sequential applications of ozone followed by phage. Microbial reductions achieved in sequential treatments were significantly higher (*P* < 0.05) than those of ozone alone treatments.

### Effect of gaseous ozone on bacteriophage OSYSP infectivity

**Figure 4:**
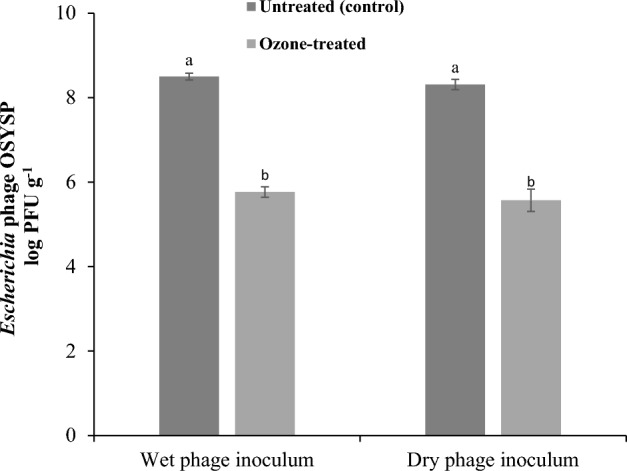
Changes in *Escherichia* phage OSYSP titer (log PFU g^−1^) on spinach leaves when gaseous ozone was applied at different phases of phage application: Ozone applied immediately after bacteriophage spray (0 h, wet phage inoculum) or 1 h after phage spray (dry phage inoculum). Average log reductions were obtained from at least 3 independent treatments. Different letters on bars represent significant difference (*P* < 0.05). Error bar indicates ± standard error of the mean.

**Figure 5:**
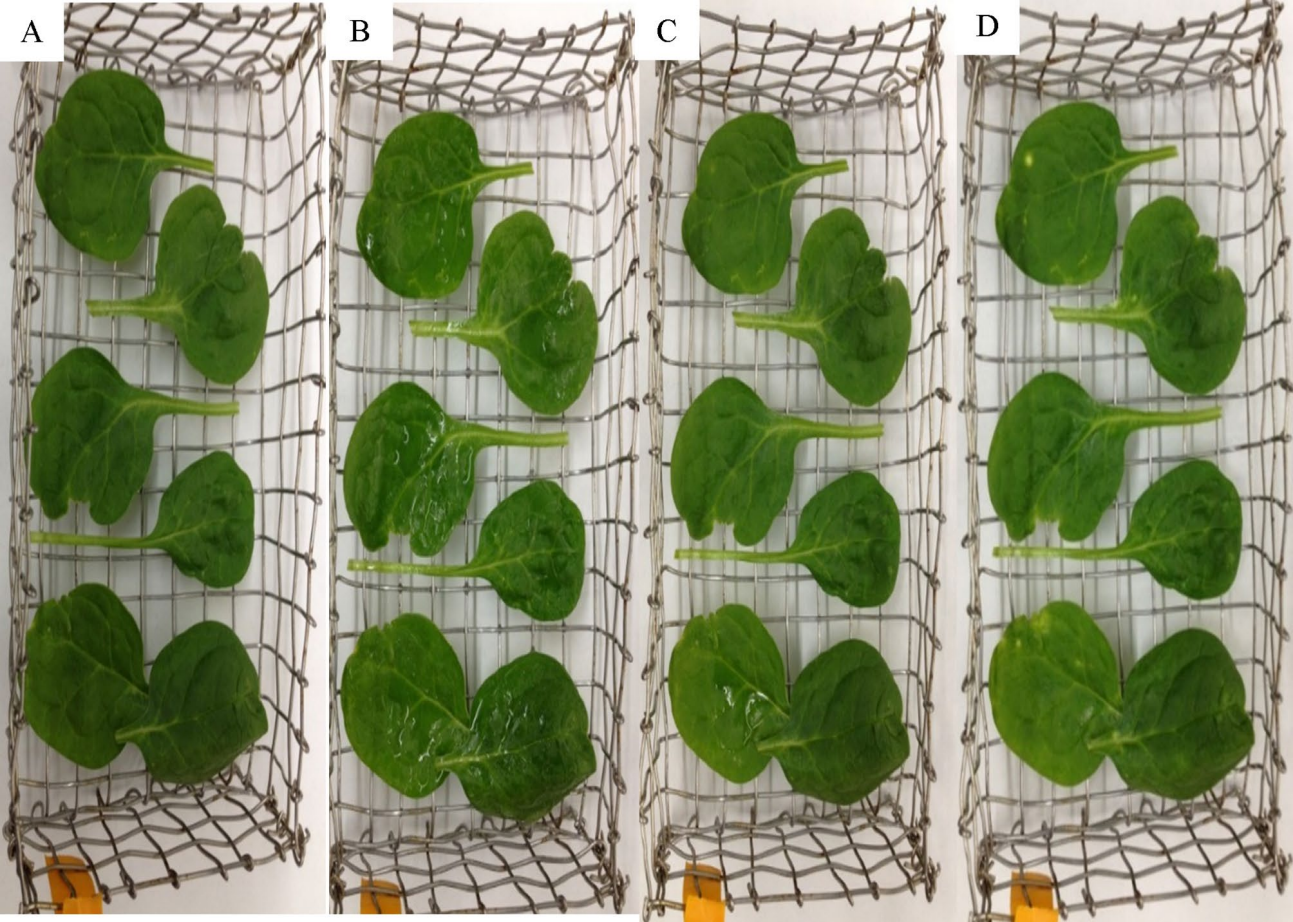
Effect of treatments on the visual quality of spinach leaves. (**A**) Untreated fresh produce, (**B**) zero-time after phage application, (**C**) one hour after phage application, (**D**) after sequential ozone-phage treatments.

Experiments were completed to evaluate the effect of gaseous ozone treatment on inactivating bacteriophage OSYSP during sequential treatments on spinach leaves (Fig. 4). When applied at zero-time (wet phage inoculum) or 1 h after phage spray (dry phage inoculum), gaseous ozone treatment reduced the bacteriophage OSYSP counts significantly (*P* < 0.05) by 2.7 log PFU g^−1^ as shown. There was no significant difference (*P* > 0.05) among the gaseous ozone treatments with respect to phage application time on spinach leaves. Additionally, phage, gaseous ozone, and their sequential treatments did not affect the visual quality of spinach leaves as observed by researchers in the study (Fig. 5).

## Discussion

The preliminary assessment of bacteriophage OSYSP efficacy against *Escherichia coli* O157:H7 revealed a 1.2 log CFU g^−1^ greater reduction in *E. coli* O157:H7 population on spinach samples treated with bacteriophage at 10^9^ PFU g^−1^, compared to 10^8^ PFU g^−1^ (Fig. 1a). A single-repeated phage spray (2 × 10^9^ PFU g^−1^) to saturate the spinach samples was not found to be significantly more effective against *E. coli* O157:H7 on spinach samples compared to the non-repeating phage spray (10^9^ PFU g^−1^) (Fig. 1b). A bacteriophage must bind to specific receptors on the susceptible host cells to initiate the infection^29^. Being extremely small in size in comparison to bacteria, bacteriophages diffuse toward their host through the surrounding liquid and encounter a permissive host randomly^30^. Subsequent adsorption of bacteriophage to the hosts is a function of chemical and physical interactions between these two entities^31^. It is hypothesized that repeating phage spray may cause better distribution of phages on the surface of spinach leaves and increase the likelihood of bacteriophages encountering their host. Although phage suspension volume may influence phage-host encounters, our findings indicated that considerable concentration increase in phage titer (i.e., tenfold or more) is necessary for exhibiting efficacy against *E. coli* O157:H7 on spinach leaves.

Application of the lytic bacteriophage as a spray decreased *E. coli* O157:H7 counts on spinach leaves by up to 2.0 log CFU g^−1^ (Fig. 2). Consistent with these findings, a previous report^17^ on the dipping *E. coli* O157:H7 contaminated spinach leaves in a suspension of the same bacteriophage, *Escherichia* phage OSYSP, for two minutes resulted in 1.4 log CFU g^−1^ decrease in the population of *E. coli* as compared to the phage-free rinsing treatment. The phage spray treatment, tested in this study, mimics the post-harvest aqueous chlorine spray on fresh produce in the field. The spray and dipping application of bacteriophages were compared in a previous study^32^. These researchers found that spraying *E. coli*-specific phages on leafy greens was more effective than dipping due to better phage particle dispersion on the leaves. In another study, applications of phage mixtures on cut lettuce leaves reduced the target population by 1.9 log CFU cm^−2^ as compared to the control treatments^33^. Similar findings have been reported by Boyacioglu et al.^34^; the researchers obtained about 2.5 log CFU cm^−2^ reduction on the target microorganism inoculated on the spinach and lettuce leaves after 30 min of phage treatments. It has been well-documented that the most widely used sanitizers react with organic matter in fresh produce wash water, thereby decreasing their efficacy against pathogenic contaminants^11,35^. Additionally, some of these reactions can lead to the formation of harmful disinfection byproducts which are detrimental to human health^36^. Unlike conventional sanitizers, bacteriophages were found to be effective biological control agents, even in an organic-rich environment^37^, with no formation of hazardous substances expected. These researchers showed that a phage cocktail in wash-water with high organic load reduced *E. coli* O157:H7 on spinach leaves by 4 log, and this efficacy was comparable to that achieved by a similar wash with the phage cocktail in sterile potable wash water^37^.

It is also noteworthy that in some cases, applying a single lytic phage could be more effective in targeting pathogens than the use of phage combinations^38^; the latter is typically done to avoid loss of activity due to the development of bacterial resistance to a phage and to extend the host range. Studies have shown that when a bacterium is simultaneously infected by dissimilar phages, mutual exclusion can occur, resulting in only one of the phages multiplying and producing progeny phages^39^. T5 phage is also found to be more effective than a phage mixture regardless of temperature and multiplicity of infection values^40^. A single lytic phage, as used in this study, with a higher concentration than its host may help prevent the emergence of bacterial resistance to phages due to the immediate lysis caused by the “lysis from without” mechanisms”^15^.

In the current study, spraying spinach leaves with a phage suspension was performed after the gaseous ozone treatments/vacuum cooling. In this antimicrobial application order, the combination treatment produced 3.4–> 4.3 log CFU g^−1^ reduction (Fig. 3), which was significantly higher than the reductions (1.7 and 2.0 log CFU g^−1^) obtained when the bacteriophage was sprayed without subsequent ozone application (Fig. 2). Synergistic intervention is achieved when the combination of antibacterial agents produces an antibacterial effect that exceeds that obtained from the combined effect of individual treatments^41^. Bacteriophage application following gaseous ozone treatment showed synergy and reduced *E. coli* O157:H7 count by more than 5 log CFU g^−1^, which was significantly higher than the sum of inactivation from individual treatments at the same inoculum size.

Utilizing bacteriophage and gaseous ozone combination can be considered a sound intervention technique against bacterial pathogens. Each of these two antimicrobials has distinct mode of action, and therefore, the two complement each other and may evade any development of bacterial resistance to the combined process. Findings from previous studies led to the conclusion that gaseous ozone damages microbial structures and sensitizes bacterial spores to a second sanitizer^42,43^. It was shown that the combination of phage cocktails with different antimicrobials increased fresh produce decontamination due to either an additional or synergistic effect when compared to the individual antimicrobial treatments^16,44^.

Enhanced antimicrobial efficacy was reported previously when bacteriophages were applied at higher concentrations than those of their hosts^16,45,46^. In the current study, enhanced effectiveness of bacteriophage OSYSP against *E. coli* O157:H7 was observed at 10^9^ PFU g^−1^ (multiplicity of infection, MOI, ≥ 100), compared to 10^8^ PFU g^−1^ (i.e., lower MOI). Pathogen inactivation on fresh produce by bacteriophages requires an initial contact of phages and their respective host microorganisms. It was suggested that high density of phage suspensions (≥ 10^9^ PFU ml^−1^) should be employed, in conjunction with excess phage in comparison to bacteria (> 100 phages per bacterium), for effective lysis-from-without mechanisms^26^. In the present study, great reductions of the pathogen were achieved after using phage preparation with high titers (≥ 10^9^ PFU ml^−1^), to achieve MOI ≥ 100. Strikingly, results of this study showed that applying gaseous ozone first reduced the pathogen level significantly; thus, phage application following ozone treatment was the most effective control strategy due to increased ratio of phage to live host-cell. It is likely that ozone treatment also helped in exposing phage receptors on host cells; this rendered them susceptible to lysis by subsequent phage application, but this hypothesis needs to be confirmed experimentally.

Reduction in different populations of *E. coli* O157:H7 on spinach leaves, obtained by gaseous ozone treatments only, ranged from 1.8 to 3.5 log CFU g^−1^. Efficacy of gaseous ozone was enhanced prominently with the decrease in pathogen’s inoculum size, a result that is in agreement with previous findings^22^. Additionally, the level of reduction in response to ozone application on contaminated spinach leaves matches that reported by Vurma et al.^47^ under similar treatment conditions. Compared to aqueous ozone, gaseous ozone was found to be more effective at eliminating microorganism due to it is greater capability to reach different locations of the leafy greens and its greater stability at the gaseous phase^48^. However, it was shown previously that oversaturation of spinach leaves with water, during a pre-washing step, resulted in antagonistic effect against the subsequent gaseous ozone treatment^49^. The sequential application of bacteriophage and gaseous ozone is envisioned as a post-harvest field treatment that can be applied in conjunction with vacuum cooling, which is applied on fresh produce before shipping to fresh-cut processing facility or distribution. The findings suggest that bacteriophage application had no adverse effect on the efficacy of the subsequent gaseous ozone treatment. Although gaseous ozone treatment achieved significant microbial reductions, population of the target microorganism was lower than the detection limit, 1 log CFU g^−1^, only when the ozone treatment preceded the bacteriophage application. Additionally, gaseous ozone inactivated 2.7 log PFU g^−1^ bacteriophage OSYSP on phage inoculated leaves. Previous research showed that residual phage that survived high temperature treatment (70 °C) was effective in inhibiting the growth of the post contaminated pathogen in milk and chicken breast^50^. Thus, further investigation is needed to determine the efficacy of residual phage OSYSP against *E. coli* O157:H7 on spinach leaves when the gaseous ozone treatment succeeds the phage application.

Color and texture are primary fresh produce traits that affect consumer preference^51^. Application of bacteriophage is not expected to change the color or texture characteristics of fresh produce^52^. However, it was reported that suspension of the phages in phosphate buffer saline showed a detrimental effect of the texture of treated leaves^34^. In a sensory study, Perrera et al.^53^ reported no adverse effect on the organoleptic features of the ready-to-eat foods after bacteriophage applications. Based on the current study, visual examination of phage-treated spinach leaves showed that the product maintained desired texture and color (Fig. 5). However, organic matter content of peptone could provide additional protection to pathogens from the ozone treatment due to organic matters’ natural reaction with ozone. Proper suspension medium for phage stock seems essential for both texture and microbial quality of the treated fresh produce. Although high gaseous ozone concentration effectively inactivates pathogens, this may be accompanied with a bleaching effect on treated fresh produce. At the conditions tested in the current study, application of gaseous ozone on the spinach leaves after the phage treatment resulted in no apparent quality deterioration (Fig. 5). These observations are consistent with those of a previous comparable ozone treatment study^47^.

It was advised that effective decontamination methods for produce should reduce the targeted pathogen by 3-log at least, whereas 5-log reduction is a goal to be achieved^54^. It is technically challenging to decontaminate fresh produce due to the lack of microbial kill-step that effectively eliminate pathogens without causing product quality deterioration. The low infectious dose of *E. coli* O157:H7 and the delicate texture of leafy greens necessitate the use of multiple mild hurdles to effectively eliminate pathogens without causing the product quality deterioration. In the current study, bacteriophage and gaseous ozone, applied individually or in combination, significantly reduced the populations of *E*. *coli* O157:H7 regardless of pathogen’s inoculum size or alternating antimicrobial application order. The sequential treatment revealed that spraying fresh produce with *Escherichia* phage OSYSP after gaseous ozone treatment/vacuum cooling would produce a synergistic antibacterial effect: thus, using the waterless sanitization step preceding the aqueous spray before packing may have practical application in fresh produce industry^12^. At the lower inoculum levels tested, the sequential treatments lowered the pathogen count below the detection limit of enumeration method while preserving the visual quality of spinach leaves. Although phage and ozone hold potential in controlling pathogens in laboratory settings, significant optimization and scale-up efforts (i.e., target strains, inoculation methods, handling produce volumes, determining the safety of phages for food use, and further optimization of phage and ozone application conditions) are needed to make these alternative approaches feasible in real-world scenarios.

## Methods

### Bacterial strains and growth conditions

*Escherichia coli* O157:H7 GFP B6-914 (*E. coli* O157:H7 B6-914) was used in the inactivation studies on fresh produce. *E. coli* O157:H7 strain B6914, also known as SEA13B88, was originally isolated from fresh apple juice associated with a disease outbreak^55^. The strain was modified to lack the expression of Shiga-toxins and to carry ampicillin resistance and green fluorescent protein genes; thus, the strain is well suited as STEC surrogate to monitor the behavior of bacteria in food and processing environment^56^. Stock culture of *E. coli* O157:H7 was kept in frozen conditions at − 80 °C. To prepare fresh culture, a loopful of *E. coli* O157:H7 from frozen culture was inoculated in Luria–Bertani (LB) broth (Becton Dickinson, Sparks, MD) supplemented with 100 μg ml^−1^ of ampicillin (Fisher scientific, Fair Lawn, NJ) and incubated overnight at 37 °C in a shaking incubator (Model; G24 Environmental incubator shaker, New Brunswick Scientific Co., Inc, Edison, NJ). This overnight incubation was followed by another transfer of *E. coli* O157:H7 B6-914 into fresh LB broth for a second overnight incubation. Thereafter, incubated LB culture was centrifuged in a benchtop centrifuge (Model; Centra MP4R, International Equipment Company) at 5500×*g* for 10 min and the cell pellet was suspended in 0.1 %, wt/vol, buffered peptone water (BPW) (Becton Dickinson). *E. coli* O157:H7 EDL 933, which was used for propagating bacteriophages, was grown under similar conditions but without the supplementation of ampicillin.

### Bacteria enumeration

*Escherichia coli* O157:H7 B6-914 populations were enumerated before and after treatments by using plate count techniques. Briefly, spinach leaves were placed in a stomacher bag and mixed with sterile 0.1%, wt/vol, of BPW. The samples were then homogenized using stomacher (Tekmar Inc., Cincinnati, OH) for two minutes and serial dilutions were prepared in BPW. To enumerate *E. coli* O157:H7 B6-914, dilutions was spread-plated on LB agar plates supplemented with 100 μg ml^−1^ of ampicillin. Colonies formed on the plates, after incubation, were enumerated under UV light. The ampicillin and green fluorescence protein genes in *E. coli* O157:H7 B6-914 makes it possible to differentiate the inoculated microorganism from the natural background microbiota of fresh produce.

### Bacteriophage stock preparation

*Escherichia* phage OSYSP was used as a single lytic biocontrol agent in this study. To cultivate phage OSYSP, pure phage suspension (10^9^ PFU ml^−1^) and host *E. coli* O157:H7 EDL 933 (10^9^ CFU ml^−1^) were mixed at a volumetric ratio of 1:100 in 20 ml LB broth, and incubated for about 4 h at 37 °C. After propagation, the mixture was centrifuged at 5500×*g* for 10 min using the benchtop centrifuge. Suspension, containing phage particles, was purified by using a 0.45-μm pore size filter (Merck Millipore Ltd, Cork, Ireland). The obtained crude phage in the supernatant was further centrifuged (58,000×*g*, 3 h) using an ultracentrifuge (Model; L8-55, Beckman Instrument, Palo Alto, CA) equipped with Type 70 Ti fixed-angle rotor. The undisturbed phage pellets were re-suspended in BPW to reach the initial phage titer of 2.5 × 10^9^ PFU ml^−1^. The purified phage stock was stored under dark conditions at 4 °C until use in experiments.

### Bacteriophage enumeration

Bacteriophage titers were determined using double agar overlay technique. To enumerate phage particles, phage suspension was diluted in BPW and mixed with 200-µl host (*E. coli* O157:H7 B6-914) suspension. The mixture of phage and host bacteria was incubated at 37 °C for 15 min to achieve the initial infection of host by phage. The incubated mixture was added to molten LB soft agar, which was held at 47 °C water bath and then overlaid on LB agar plates. Plates were incubated overnight at 37 °C to determine the phage titer. For matrices containing phage, the sample was homogenized, filtered, and concentration of phage in filtrate was determined using double agar overlay method as described above.

### Inoculation of spinach leaves

Spinach leaves were purchased from a local market (Columbus, Ohio, USA) at the same day of experiments and stored at 4 °C in a cooler prior to each experiment. Spinach leaves were divided into perforated sample trays, each of which contained approximately 5 g of spinach (approximately 5 whole leaves). Leaves were then inoculated with several spots of the pathogen cell suspension on surfaces (spot-inoculation), which yielded initial populations of 10^5^–10^7^ *E. coli* O157:H7 B6-914 CFU g^−1^; different populations were used in different experiments. The inoculated leaves were air-dried for an hour in a laminar-flow biosafety cabinet (Model; Class II Type A/B3, NuAire Inc, Plymouth, MN) at 20 °C for bacterial attachment before phage and ozone treatments.

### Bacteriophage application on spinach leaves

Spray bottles were utilized to deliver the bacteriophage suspensions on spinach leaves. To determine the effective phage concentration on *E. coli* O157:H7 B6-914 inactivation, 20 g of inoculated spinach leaves in four trays (approximate initial load of 10^7^ CFU g^−1^) were sprayed with bacteriophage OSYSP (0.5 ml per gram of spinach) at the concentrations of 2.5 × 10^8^ or 2.5 × 10^9^ PFU ml^−1^. Control samples were inoculated with *E. coli* O157:H7 B6-914 but sprayed with phage-free BPW instead. To determine the effect of spray volume of spinach leaves with the antibacterial phage suspension on pathogen inactivation, a single spray of bacteriophage OSYSP (0.5 ml per gram of spinach) was applied immediately following the initial phage application, resulting in a final volume of 1 ml of bacteriophage OSYSP per gram of spinach sample. Treated leaves were held for one hour in a laminar flow biological hood (Model; Class II Type A/B3, NuAire Inc, Plymouth, Mn) at 20 °C before enumerations.

### Gaseous ozone treatment conditions

**Figure 6:**
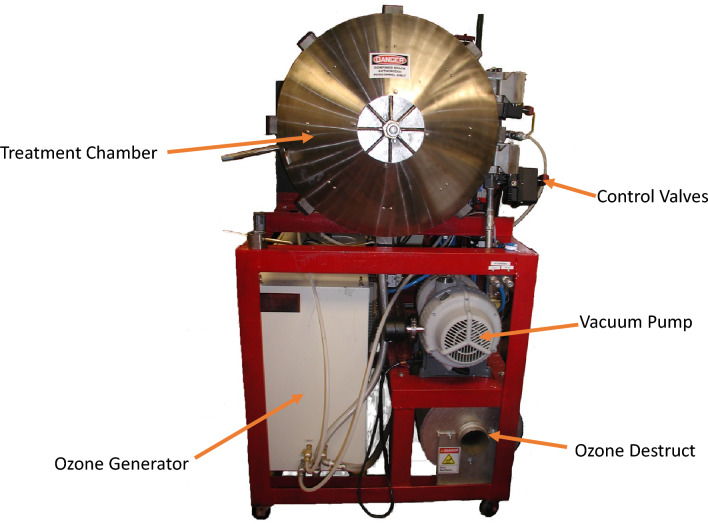
Experimental apparatus and parts used for gaseous ozone treatment of spinach leaves during vacuum cooling.

**Figure 7:**
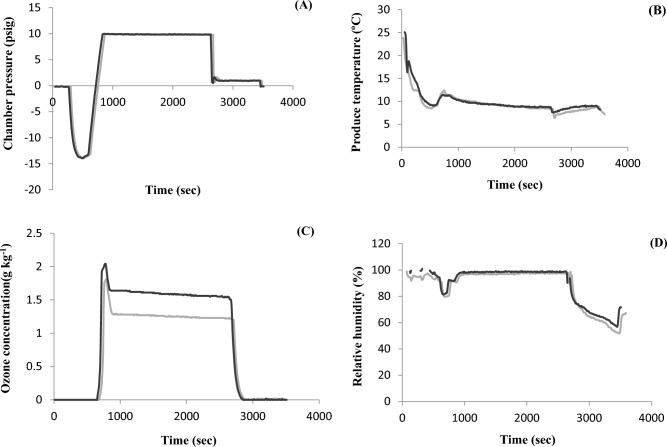
Critical process parameters of the gaseous ozone treatment during vacuum cooling; presented are graphs from two separate experimental runs, shown in black and gray. (**A**) Ozone treatment chamber pressure, (**B**) temperature of spinach leaves, (**C**) gaseous ozone concentration, and (**D**) relative humidity in the ozone treatment chamber.

To treat spinach samples with gaseous ozone, a maximum of 4 trays (20 g) of spinach leaves were placed in a custom design 300-l stainless steel treatment vessel (Fig. 6). The treatment vessel was cooled using a chiller (Model NESLAB RTE 10, Thermo Electron Corporation, Newington, NH) prior to gaseous ozone treatments. The actual process was started with the application of vacuum using a dry vacuum pump (Model XDS35i, Edwards limited, West Sussex, UK) to achieve 28.5 in. Hg and simulate the industrial vacuum cooling of fresh produce. When the desired vacuum was achieved, the vessel was pressurized to 10 psig with gaseous ozone-oxygen gas mix to achieve a concentration of 1.5 g ozone/kg gas-mix. During the vacuum cooling process, and before the vessel pressure reached atmospheric pressure, water was sprayed into the vessel to maintain 95–100% relative humidity level during the process. Ozone gas was produced from a high-capacity ozone generator (Ozat CFS-7 2G; Ozonia Inc., Elmwood Park, NJ). Ozone concentration during processing was monitored by high and low-capacity ozone monitors (Models 454 M and 454H, Teledyne Instruments, San Diego, CA) at a wavelength of 254 nm. After 30 min holding time, the gaseous ozone that remained in the vessel was sent to a thermal destruct unit at 235 °C for decomposition of the gaseous ozone to oxygen. This exhaust process was completed by flushing the vessel with air for 15 min to achieve complete removal of excessive ozone. After ozone gas inside the vessel was exhausted, the process was completed, and the treated samples were taken out for microbial analysis. Critical process parameters monitored and recorded were shown in Fig. 7.

### Sequential treatments of inoculated spinach leaves with bacteriophage followed by gaseous ozone

Thirty grams of inoculated spinach leaves (approximate initial loads; 10^5^ or 10^7^ CFU g^−1^) were sprayed with 2.5 × 10^9^ PFU ml^−1^ *Escherichia* phage OSYSP (0.5 ml per gram of spinach). Control treatment leaves (10 g) were inoculated with target microorganisms but treated with phage-free BPW instead. Phage-treated leaves were held for an hour in a laminar flow biological hood (NuAire Inc, Plymouth) at 20 °C before sequential treatment with gaseous ozone for 30 min of holding time at 1.5 g ozone in a kg gas-mix at 10 psig holding pressure, as described above.

### Sequential treatments of inoculated spinach leaves with gaseous ozone followed by bacteriophage

Twenty grams of spot-inoculated spinach leaves (approximate initial loads; 10^5^ or 10^7^ CFU g^−1^) were initially subjected to a gaseous ozone treatment during vacuum cooling under the conditions described previously. Immediately after the completion of the gaseous ozone treatment, half of the ozone treated leaves were used to enumerate the efficacy of gaseous ozone treatments to inactivate *E. coli* O157:H7 B6-914 on spinach leaves, whereas the remaining ozone-treated leaves were sprayed with *Escherichia* phage OSYSP at ~ 10^9^ PFU ml^−1^ (0.5 ml per gram of spinach) and held for 5 min in a laminar flow biological hood (NuAire Inc, Plymouth) at 20 °C to evaluate the efficacy of sequential treatments.

### Effect of gaseous ozone on *Escherichia* phage OSYSP infectivity

The effect of the gaseous ozone application was evaluated to assess the fate of bacteriophages, which was applied on fresh produce prior to the gaseous ozone in sequential treatments. Spinach leaves were inoculated with *E. coli* O157:H7 B6-914 and sprayed with bacteriophage OSYSP (0.5 ml per gram of spinach). Phage-treated leaves were immediately (wet phage inoculum), or 1 h after phage spray (dry phage inoculum), treated with gaseous ozone during vacuum cooling, as described previous. Phage titers were determined using double agar overlay technique.

### Data analysis

There were at least three independent experiments, with two sample trays per replicate, for each treatment. Average microbial reductions from experiments were converted to logarithmic values and subjected to statistical analyses. Statistical analyses of data were carried out by using SAS PROC GLM version 9.4. Comparison of the significant differences among the groups were performed using least square means function. Differences at *P* < 0.05 was considered significant.

### Ethical approval

The use of plant parts in the study complies with international, national, and/or institutional guidelines.

## Data Availability

All data generated or analyzed during this study are included in the article.
